# 2-Chloro-*N*-(3,5-dimethyl­phen­yl)benzamide

**DOI:** 10.1107/S1600536809001706

**Published:** 2009-02-04

**Authors:** B. Thimme Gowda, Sabine Foro, B. P. Sowmya, Hartmut Fuess

**Affiliations:** a Department of Chemistry, Mangalore University, Mangalagangotri 574 199, Mangalore, India; b Institute of Materials Science, Darmstadt University of Technology, Petersenstrasse 23, D-64287 Darmstadt, Germany

## Abstract

In the structure of the the title compound, C_15_H_14_ClNO, the N—H and C=O bonds are *trans* to each other and the amide O atom is *anti* to the *ortho*-Cl atom in the benzoyl ring. The amide group makes dihedral angles of 61.2 (6) and 42.2 (8)° with the benzoyl and aniline rings, respectively. In the crystal, the mol­ecules are linked into infinite chains by N—H⋯O hydrogen bonds.

## Related literature

For the synthesis, see: Gowda *et al.* (2003 ▶). For structure of the 3,5-dichloro­phenyl analog and other benzanilides, see: Gowda *et al.* (2008**a* ▶,b* ▶).
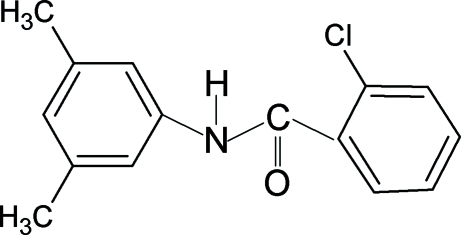


## Experimental

### 

#### Crystal data

C_15_H_14_ClNO
*M* *_r_* = 259.72
Orthorhombic, 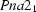

*a* = 9.1867 (6) Å
*b* = 13.9710 (8) Å
*c* = 10.2711 (7) Å
*V* = 1318.27 (15) Å^3^
*Z* = 4
Mo *K*α radiation
μ = 0.28 mm^−1^
*T* = 100 (2) K
0.48 × 0.28 × 0.13 mm

#### Data collection

Oxford Diffraction Xcalibur diffractometer with a Sapphire CCD detector
Absorption correction: multi-scan (*CrysAlis RED*; Oxford Diffraction, 2007 ▶) *T*_min_ = 0.879, *T*_max_ = 0.965
6034 measured reflections
2136 independent reflections
1977 reflections with *I* > 2σ(*I*)
*R*_int_ = 0.014

#### Refinement

*R*[*F* ^2^ > 2σ(*F* ^2^)] = 0.031
*wR*(*F* ^2^) = 0.083
*S* = 1.03
2136 reflections
187 parameters
2 restraints
H atoms treated by a mixture of independent and constrained refinement
Δρ_max_ = 0.34 e Å^−3^
Δρ_min_ = −0.27 e Å^−3^
Absolute structure: Flack (1983 ▶), 712 Friedel pairs
Flack parameter: 0.01 (7)

### 

Data collection: *CrysAlis CCD* (Oxford Diffraction, 2007 ▶); cell refinement: *CrysAlis RED* (Oxford Diffraction, 2007 ▶); data reduction: *CrysAlis RED*; program(s) used to solve structure: *SHELXS97* (Sheldrick, 2008 ▶); program(s) used to refine structure: *SHELXL97* (Sheldrick, 2008 ▶); molecular graphics: *PLATON* (Spek, 2003 ▶); software used to prepare material for publication: *SHELXL97*.

## Supplementary Material

Crystal structure: contains datablocks I, global. DOI: 10.1107/S1600536809001706/ng2536sup1.cif

Structure factors: contains datablocks I. DOI: 10.1107/S1600536809001706/ng2536Isup2.hkl

Additional supplementary materials:  crystallographic information; 3D view; checkCIF report

## Figures and Tables

**Table 1:** Hydrogen-bond geometry (Å, °)

*D*—H⋯*A*	*D*—H	H⋯*A*	*D*⋯*A*	*D*—H⋯*A*
N1—H1N⋯O1^i^	0.831 (17)	2.120 (18)	2.918 (2)	161 (2)

Symmetry code: (i) 
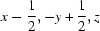
.

**Fig. 1.**
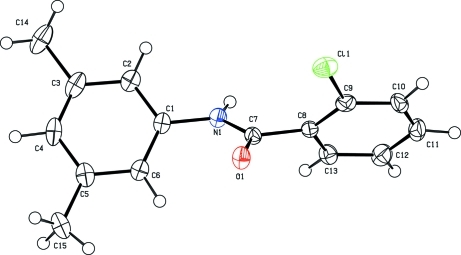
Molecular structure of the title compound, showing the atom labeling scheme. The displacement ellipsoids are drawn at the 50% probability level. H atoms are represented as small spheres of arbitrary radii.

**Fig. 2.**
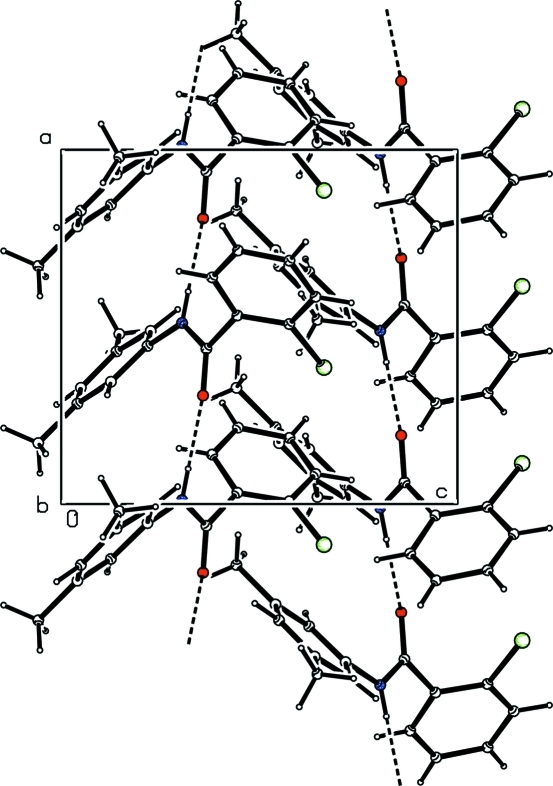
Molecular packing of the title compound with hydrogen bonding shown as dashed lines.

## supplementary crystallographic information

### Comment

In the present work, the structure of 2-chloro-*N*-(3,5-dimethylphenyl)- benzamide (N35DMP2CBA) has been determined to explore the substituent effects on the solid state structures of benzanilides (Gowda *et al.*, 2003, 2008*a,b*). The conformations of N—H and C=O bonds in the amide group of N35DMP2CBA are *trans* to each other (Fig.1), similar to that observed in 2-chloro-*N*-(3,5-dichlorophenyl)-benzamide(N35DCP2CBA) (Gowda *et al.*, 2008*a*), 2-chloro-*N*-(phenyl)-benzamide (NP2CBA) (Gowda *et al.*, 2003) and other benzanilides (Gowda *et al.*, 2008*b*). Further, the conformation of the amide oxygen in N35DMP2CBA is *anti* to the *ortho*-chloro group in the benzoyl ring similar to that observed in N35DCP2CBA but in contrast to the *syn* conformation observed in NP2CBA. The amide group –NHCO– makes the dihedral angles of 61.2 (6)° and 42.2 (8)° with the benzoyl and aniline rings, respectively, while the benzoyl and aniline rings form the dihedral angle of 76.7 (1)°), compared to the corresponding values of 63.1 (12)°, 31.1 (17)° and 32.1 (2)°) in N35DCP2CBA. Part of the crystal structure of the title compound with infinite molecular chains running along the *a* axis is shown in Fig. 2. The chains are generated by N—H···O hydrogen bonds (Table 1)

### Experimental

The title compound was prepared according to the literature method (Gowda *et al.*, 2003). The purity of the compound was checked by determining its melting point. It was characterized by recording its infrared and NMR spectra. Single crystals of the title compound were obtained from an ethanolic solution and used for X-ray diffraction studies at room temperature.

### Refinement

The H atoms of the methyl groups were positioned with idealized geometry using a riding model with C—H = 0.98 Å. The other H atoms were located in difference map, and its positional parameters were refined freely with C—H = 0.89 (3)–0.99 (3) Å, while the H atom of the NH group was later restrained to the distance 0.86 (2) Å. All H atoms were refined with isotropic displacement parameters (set to 1.2 times of the *U*_eq_ of the parent atom).

### Crystal data

**Table:**

C_15_H_14_ClNO	*F*(000) = 544
*M**_r_* = 259.72	*D*_x_ = 1.309 Mg m^−^^3^
Orthorhombic, *P**n**a*2_1_	Mo *K*α radiation, λ = 0.71073 Å
Hall symbol: P 2c -2n	Cell parameters from 2104 reflections
*a* = 9.1867 (6) Å	θ = 2.5–28.1°
*b* = 13.9710 (8) Å	µ = 0.28 mm^−^^1^
*c* = 10.2711 (7) Å	*T* = 100 K
*V* = 1318.27 (15) Å^3^	Prism, colourless
*Z* = 4	0.48 × 0.28 × 0.13 mm

### Data collection

**Table:**

Oxford Diffraction Xcalibur diffractometer with a Sapphire CCD detector	2136 independent reflections
Radiation source: fine-focus sealed tube	1977 reflections with *I* > 2σ(*I*)
graphite	*R*_int_ = 0.014
Rotation method data acquisition using ω and φ scans	θ_max_ = 26.4°, θ_min_ = 2.5°
Absorption correction: multi-scan (*CrysAlis RED*; Oxford Diffraction, 2007)	*h* = −11→11
*T*_min_ = 0.879, *T*_max_ = 0.965	*k* = −17→17
6034 measured reflections	*l* = −10→12

### Refinement

**Table:**

Refinement on *F*^2^	Secondary atom site location: difference Fourier map
Least-squares matrix: full	Hydrogen site location: inferred from neighbouring sites
*R*[*F*^2^ > 2σ(*F*^2^)] = 0.031	H atoms treated by a mixture of independent and constrained refinement
*wR*(*F*^2^) = 0.083	*w* = 1/[σ^2^(*F*_o_^2^) + (0.0477*P*)^2^ + 0.598*P*] where *P* = (*F*_o_^2^ + 2*F*_c_^2^)/3
*S* = 1.02	(Δ/σ)_max_ = 0.002
2136 reflections	Δρ_max_ = 0.34 e Å^−^^3^
187 parameters	Δρ_min_ = −0.27 e Å^−^^3^
2 restraints	Absolute structure: Flack (1983), 712 Friedel pairs
Primary atom site location: structure-invariant direct methods	Flack parameter: 0.01 (7)

### Special details

**Table:**

Geometry. All e.s.d.'s (except the e.s.d. in the dihedral angle between two l.s. planes) are estimated using the full covariance matrix. The cell e.s.d.'s are taken into account individually in the estimation of e.s.d.'s in distances, angles and torsion angles; correlations between e.s.d.'s in cell parameters are only used when they are defined by crystal symmetry. An approximate (isotropic) treatment of cell e.s.d.'s is used for estimating e.s.d.'s involving l.s. planes.
Refinement. Refinement of *F*^2^ against ALL reflections. The weighted *R*-factor *wR* and goodness of fit *S* are based on *F*^2^, conventional *R*-factors *R* are based on *F*, with *F* set to zero for negative *F*^2^. The threshold expression of *F*^2^ > σ(*F*^2^) is used only for calculating *R*-factors(gt) *etc*. and is not relevant to the choice of reflections for refinement. *R*-factors based on *F*^2^ are statistically about twice as large as those based on *F*, and *R*- factors based on ALL data will be even larger.

### Fractional atomic coordinates and isotropic or equivalent isotropic displacement parameters (Å^2^)

**Table:**

	*x*	*y*	*z*	*U*_iso_*/*U*_eq_	
Cl1	0.11485 (6)	0.32674 (4)	1.16333 (7)	0.03305 (16)	
O1	0.19372 (16)	0.32548 (10)	0.85807 (18)	0.0253 (4)	
N1	−0.01100 (18)	0.24094 (12)	0.8052 (2)	0.0210 (4)	
H1N	−0.0995 (19)	0.2359 (18)	0.820 (3)	0.025*	
C1	0.0540 (2)	0.17351 (13)	0.7178 (2)	0.0211 (4)	
C2	0.0112 (2)	0.07840 (15)	0.7257 (3)	0.0247 (5)	
H2	−0.049 (3)	0.0577 (18)	0.794 (3)	0.030*	
C3	0.0694 (2)	0.01080 (14)	0.6398 (3)	0.0299 (6)	
C4	0.1719 (3)	0.04035 (16)	0.5498 (3)	0.0307 (5)	
H4	0.219 (3)	−0.0046 (18)	0.489 (3)	0.037*	
C5	0.2166 (3)	0.13617 (16)	0.5410 (2)	0.0287 (5)	
C6	0.1549 (2)	0.20260 (15)	0.6256 (2)	0.0249 (5)	
H6	0.192 (3)	0.2682 (18)	0.617 (3)	0.030*	
C7	0.0614 (2)	0.31152 (14)	0.8664 (2)	0.0200 (4)	
C8	−0.0321 (2)	0.37827 (13)	0.9446 (2)	0.0193 (4)	
C9	−0.0080 (2)	0.39557 (15)	1.0757 (2)	0.0237 (5)	
C10	−0.0849 (3)	0.46637 (16)	1.1420 (2)	0.0289 (5)	
H10	−0.068 (3)	0.4734 (19)	1.232 (3)	0.035*	
C11	−0.1868 (2)	0.52064 (16)	1.0761 (3)	0.0313 (6)	
H11	−0.240 (3)	0.5734 (18)	1.117 (3)	0.038*	
C12	−0.2157 (3)	0.50321 (15)	0.9463 (3)	0.0305 (5)	
H12	−0.285 (3)	0.539 (2)	0.907 (3)	0.037*	
C13	−0.1396 (2)	0.43181 (16)	0.8809 (3)	0.0239 (5)	
H13	−0.157 (3)	0.4197 (18)	0.798 (3)	0.029*	
C14	0.0200 (3)	−0.09234 (15)	0.6467 (4)	0.0409 (7)	
H14A	0.0078	−0.1111	0.7380	0.049*	
H14B	0.0932	−0.1335	0.6057	0.049*	
H14C	−0.0729	−0.0993	0.6008	0.049*	
C15	0.3281 (3)	0.16747 (18)	0.4422 (3)	0.0392 (6)	
H15A	0.2854	0.1645	0.3548	0.047*	
H15B	0.4128	0.1250	0.4465	0.047*	
H15C	0.3585	0.2333	0.4610	0.047*	

### Atomic displacement parameters (Å^2^)

**Table:**

	*U*^11^	*U*^22^	*U*^33^	*U*^12^	*U*^13^	*U*^23^
Cl1	0.0362 (3)	0.0325 (3)	0.0304 (3)	−0.0048 (2)	−0.0079 (3)	0.0058 (3)
O1	0.0141 (7)	0.0272 (7)	0.0345 (10)	−0.0016 (5)	0.0019 (7)	−0.0081 (7)
N1	0.0133 (8)	0.0218 (8)	0.0280 (10)	−0.0020 (6)	0.0015 (8)	−0.0034 (7)
C1	0.0164 (9)	0.0219 (10)	0.0249 (11)	0.0019 (7)	−0.0043 (9)	−0.0049 (8)
C2	0.0178 (9)	0.0242 (10)	0.0323 (13)	−0.0008 (8)	−0.0045 (10)	−0.0028 (9)
C3	0.0219 (10)	0.0225 (9)	0.0454 (16)	0.0042 (7)	−0.0137 (11)	−0.0063 (10)
C4	0.0269 (11)	0.0306 (11)	0.0345 (14)	0.0099 (9)	−0.0078 (11)	−0.0150 (10)
C5	0.0261 (11)	0.0337 (11)	0.0262 (14)	0.0060 (9)	−0.0032 (11)	−0.0064 (10)
C6	0.0238 (10)	0.0221 (10)	0.0286 (13)	0.0010 (8)	−0.0015 (9)	−0.0030 (8)
C7	0.0180 (10)	0.0211 (9)	0.0208 (12)	−0.0005 (7)	0.0029 (9)	−0.0011 (8)
C8	0.0172 (9)	0.0188 (9)	0.0220 (12)	−0.0048 (7)	0.0034 (9)	−0.0019 (8)
C9	0.0211 (9)	0.0228 (10)	0.0273 (13)	−0.0085 (8)	0.0010 (9)	0.0008 (8)
C10	0.0351 (12)	0.0282 (10)	0.0235 (14)	−0.0127 (8)	0.0098 (11)	−0.0094 (9)
C11	0.0271 (11)	0.0249 (10)	0.0418 (16)	−0.0037 (8)	0.0135 (11)	−0.0097 (10)
C12	0.0221 (11)	0.0270 (11)	0.0424 (16)	0.0016 (9)	0.0052 (12)	−0.0005 (10)
C13	0.0188 (10)	0.0279 (10)	0.0250 (13)	−0.0015 (8)	0.0016 (9)	−0.0032 (9)
C14	0.0319 (12)	0.0227 (10)	0.068 (2)	0.0007 (8)	−0.0118 (14)	−0.0102 (13)
C15	0.0412 (14)	0.0471 (14)	0.0294 (14)	0.0052 (12)	0.0090 (13)	−0.0100 (12)

### Geometric parameters (Å, °)

**Table:**

Cl1—C9	1.735 (2)	C8—C9	1.386 (3)
O1—C7	1.234 (3)	C8—C13	1.401 (3)
N1—C7	1.346 (3)	C9—C10	1.393 (3)
N1—C1	1.431 (3)	C10—C11	1.383 (4)
N1—H1N	0.831 (17)	C10—H10	0.94 (3)
C1—C6	1.386 (3)	C11—C12	1.380 (4)
C1—C2	1.388 (3)	C11—H11	0.98 (3)
C2—C3	1.399 (3)	C12—C13	1.391 (3)
C2—H2	0.94 (3)	C12—H12	0.91 (3)
C3—C4	1.382 (4)	C13—H13	0.89 (3)
C3—C14	1.512 (3)	C14—H14A	0.9800
C4—C5	1.403 (3)	C14—H14B	0.9800
C4—H4	0.99 (3)	C14—H14C	0.9800
C5—C6	1.392 (3)	C15—H15A	0.9800
C5—C15	1.506 (4)	C15—H15B	0.9800
C6—H6	0.98 (3)	C15—H15C	0.9800
C7—C8	1.501 (3)		
			
C7—N1—C1	124.70 (17)	C8—C9—C10	121.2 (2)
C7—N1—H1N	117.2 (19)	C8—C9—Cl1	120.74 (16)
C1—N1—H1N	118.1 (19)	C10—C9—Cl1	118.02 (19)
C6—C1—C2	120.7 (2)	C11—C10—C9	119.5 (2)
C6—C1—N1	120.94 (18)	C11—C10—H10	122.3 (18)
C2—C1—N1	118.4 (2)	C9—C10—H10	118.1 (18)
C1—C2—C3	120.1 (2)	C12—C11—C10	120.4 (2)
C1—C2—H2	120.5 (16)	C12—C11—H11	116.7 (18)
C3—C2—H2	119.2 (16)	C10—C11—H11	122.8 (18)
C4—C3—C2	118.7 (2)	C11—C12—C13	119.8 (2)
C4—C3—C14	121.3 (2)	C11—C12—H12	118 (2)
C2—C3—C14	119.9 (2)	C13—C12—H12	122 (2)
C3—C4—C5	121.8 (2)	C12—C13—C8	120.7 (2)
C3—C4—H4	122.2 (16)	C12—C13—H13	120.8 (17)
C5—C4—H4	116.0 (16)	C8—C13—H13	118.5 (17)
C6—C5—C4	118.4 (2)	C3—C14—H14A	109.5
C6—C5—C15	120.3 (2)	C3—C14—H14B	109.5
C4—C5—C15	121.3 (2)	H14A—C14—H14B	109.5
C1—C6—C5	120.2 (2)	C3—C14—H14C	109.5
C1—C6—H6	124.8 (16)	H14A—C14—H14C	109.5
C5—C6—H6	114.9 (16)	H14B—C14—H14C	109.5
O1—C7—N1	124.76 (19)	C5—C15—H15A	109.5
O1—C7—C8	120.18 (18)	C5—C15—H15B	109.5
N1—C7—C8	115.01 (18)	H15A—C15—H15B	109.5
C9—C8—C13	118.23 (19)	C5—C15—H15C	109.5
C9—C8—C7	122.49 (19)	H15A—C15—H15C	109.5
C13—C8—C7	119.0 (2)	H15B—C15—H15C	109.5
			
C7—N1—C1—C6	−42.6 (3)	O1—C7—C8—C9	−57.9 (3)
C7—N1—C1—C2	138.7 (2)	N1—C7—C8—C9	124.6 (2)
C6—C1—C2—C3	−0.3 (3)	O1—C7—C8—C13	116.2 (2)
N1—C1—C2—C3	178.36 (19)	N1—C7—C8—C13	−61.3 (3)
C1—C2—C3—C4	1.4 (3)	C13—C8—C9—C10	−2.1 (3)
C1—C2—C3—C14	−178.6 (2)	C7—C8—C9—C10	172.08 (18)
C2—C3—C4—C5	−1.2 (4)	C13—C8—C9—Cl1	175.77 (15)
C14—C3—C4—C5	178.8 (2)	C7—C8—C9—Cl1	−10.1 (3)
C3—C4—C5—C6	−0.2 (4)	C8—C9—C10—C11	−0.2 (3)
C3—C4—C5—C15	−179.8 (2)	Cl1—C9—C10—C11	−178.07 (16)
C2—C1—C6—C5	−1.1 (3)	C9—C10—C11—C12	1.9 (3)
N1—C1—C6—C5	−179.7 (2)	C10—C11—C12—C13	−1.3 (3)
C4—C5—C6—C1	1.3 (3)	C11—C12—C13—C8	−1.1 (3)
C15—C5—C6—C1	−179.0 (2)	C9—C8—C13—C12	2.7 (3)
C1—N1—C7—O1	−2.2 (4)	C7—C8—C13—C12	−171.68 (19)
C1—N1—C7—C8	175.16 (19)		

### Hydrogen-bond geometry (Å, °)

**Table:**

*D*—H···*A*	*D*—H	H···*A*	*D*···*A*	*D*—H···*A*
N1—H1N···O1^i^	0.83 (2)	2.12 (2)	2.918 (2)	161 (2)

Symmetry codes: (i) *x*−1/2, −*y*+1/2, *z*.

## References

[bb1] Flack, H. D. (1983). *Acta Cryst.* A**39**, 876–881.

[bb2] Gowda, B. T., Foro, S., Sowmya, B. P. & Fuess, H. (2008*a*). *Acta Cryst.* E**64**, o1294. 10.1107/S1600536808018072 PMC2961721 21202924

[bb3] Gowda, B. T., Jyothi, K., Paulus, H. & Fuess, H. (2003). *Z. Naturforsch. Teil A*, **58**, 225–230.

[bb4] Gowda, B. T., Tokarčík, M., Kožíšek, J., Sowmya, B. P. & Fuess, H. (2008*b*). *Acta Cryst.* E**64**, o1365. 10.1107/S1600536808019120 PMC2961789 21202983

[bb5] Oxford Diffraction (2007). *CrysAlis CCD* and *CrysAlis RED* Oxford Diffraction Ltd, Abingdon, England.

[bb6] Sheldrick, G. M. (2008). *Acta Cryst.* A**64**, 112–122. 10.1107/S0108767307043930 18156677

[bb7] Spek, A. L. (2003). *J. Appl. Cryst.* **36**, 7–13.

